# Musculoskeletal simulation of professional ski jumpers during take-off considering aerodynamic forces

**DOI:** 10.3389/fbioe.2023.1241135

**Published:** 2023-08-31

**Authors:** Yi Huang, Liang Jiang, Xue Chen, Qing Sun, Xiao Zhang, Xunan Tan, Yan Du, Fangtong Zhang, Nannan Wang, Rufeng Su, Feng Qu, Guoqing Zhang, Bo Huo

**Affiliations:** ^1^ School of Aerospace Engineering, Beijing Institute of Technology, Beijing, China; ^2^ Biomechanics Laboratory, Beijing Sport University, Beijing, China; ^3^ Institute of Artificial Intelligence in Sports, Capital University of Physical Education and Sports, Beijing, China

**Keywords:** ski jumping, take-off, musculoskeletal simulation, aerodynamics, muscle activation, joint torque, ground reaction force

## Abstract

**Introduction:** Musculoskeletal simulation has been widely used to analyze athletes’ movements in various competitive sports, but never in ski jumping. Aerodynamic forces during ski jumping take-off have been difficult to account for in dynamic simulation. The purpose of this study was to establish an efficient approach of musculoskeletal simulation of ski jumping take-off considering aerodynamic forces and to analyze the muscle function and activity.

**Methods:** Camera-based marker-less motion capture was implemented to measure the take-off kinematics of eight professional jumpers. A suitable full-body musculoskeletal model was constructed for the simulation. A method based on inverse dynamics iteration was developed and validated to estimate the take-off ground reaction force. The aerodynamic forces, which were calculated based on body kinematics and computational fluid dynamics simulations, were exerted on the musculoskeletal model as external forces. The activation and joint torque contributions of lower extremity muscles were calculated through static optimization.

**Results:** The estimated take-off ground reaction forces show similar trend with the results from past studies. Although overall inconsistencies between simulated muscle activation and EMG from previous studies were observed, it is worth noting that the activation of the tibialis anterior, gluteus maximus, and long head of the biceps femoris was similar to specific EMG results. Among lower extremity extensors, soleus, vastus lateralis, biceps femoris long head, gluteus maximus, and semimembranosus showed high levels of activation and joint extension torque contribution.

**Discussion:** Results of this study advanced the understanding of muscle action during ski jumping take-off. The simulation approach we developed may help guide the physical training of jumpers for improved take-off performance and can also be extended to other phases of ski jumping.

## 1 Introduction

Ski jumping has been one of the competitive disciplines in the very first Olympic Winter Game in 1924 (Braghin et al., 2016). The ski jumping movement sequence is divided into the following four phases: in-run, take-off, flight, and landing. Among these phases, take-off has been considered the most important one as it determines the initial state of the flight phase (Chardonnens et al., 2014).

Previous biomechanical studies on take-off have been mainly focused on body kinematics and dynamics. Throughout the take-off, body velocity, acceleration, angular velocity, angular momentum, energy, and joint torque were reported, among which the acceleration at the center of mass and forward somersaulting angular velocity were important for performance (Arndt et al., 1995; Sasaki et al., 1997; Sasaki et al., 2005; Virmavirta and Komi, 2010; Chardonnens et al., 2014). Some other studies have been carried out on ground reaction force (GRF). Measuring devices such as force plate installed on the ski jumping platform or insole pressure sensors were once used, but never popularized due to their high cost (Virmavirta and Koml, 1993a; b; Virmavirta et al., 2001). Later studies focused more on data-driven GRF estimation methods (Fritz et al., 2019; Nam et al., 2023). Although these studies have revealed the correlation of take-off kinematics and dynamics with athletes’ performance, the activity and function of lower extremity muscle during take-off have not yet been fully elucidated.

Aerodynamics is another vital factor in ski jumping. Past aerodynamic studies have focused more on the flight phase of ski jumping because of the significant aerodynamic effects involved (Barnes et al., 2022; Yamamoto et al., 2022). The role of aerodynamics during take-off has not been studied extensively (Braghin et al., 2016). Virmavirta et al. (Virmavirta et al., 2011) have compared take-off characteristic under nonwind and wind condition through wind tunnel experiment. Differences in body posture, take-off time and GRF between the two conditions were reported. Yamamoto et al. reported an aerodynamic resultant force of over 100 N at the end of take-off (Yamamoto et al., 2016), which could bring a substantial impact on body dynamics of jumpers. However, aerodynamic effects have rarely been considered in previous studies of take-off dynamics.

Electromyography (EMG) has been the most commonly used approach to study muscle activation patterns. However, there are only a few EMG studies on hill jumps with a limited number of muscles that are measured. Virmavirta et al. (Virmavirta et al., 2001; Virmavirta and Komi, 2001) have reported the EMG results of several lower body muscles during take-off. Their studies indicated that the gluteus maximus and vastus lateralis are major contributors to the take-off, while ankle plantar flexors play a limited role. There might be two reasons for the limitation of such studies. Firstly, the large displacement of jumpers during hill jumps brings difficulties to the signal transmission and reception of the EMG sensor. Secondly, athletes’ movements can be severely affected when equipped with EMG sensors, and the risk of injury may therefore increase.

Human locomotion simulation based on musculoskeletal model is another practical approach for estimating muscle state, which is useful for athletes’ physical training, motion optimization, and injury risk prediction. This technique has been applied to various competitive sports, such as basketball (Akhundov et al., 2022), golf (Mahadas et al., 2019), cross-country sit-skiing (Chen et al., 2023), swimming (Langholz et al., 2016), etc. Musculoskeletal simulation of ski jumping take-off has not yet been reported so far, possibly due to the difficulty of kinematic and external forces (GRF and aerodynamic force) acquisition in-field. However, these difficulties are expected to be overcome in view of recent research advances. The accuracy of vision-based marker-less motion capture methods has continued to improve with advances in AI and computer vision. Drazan et al. verified the consistency between the kinematics derived from marker-less motion-capture and marker-based motion capture during vertical jumping (Drazan et al., 2021). Further, Nam et al. predicted a GRF close to the measured one based on the kinematics acquired by a marker-less motion capture system and applied it to in-field take-off (Nam et al., 2023). In terms of aerodynamics, although it is difficult to measure the aerodynamics of a specific jump directly, researchers have established a method to estimate posture-corresponding aerodynamic forces based on a series of wind tunnel test results. Jung et al. have performed ballistics studies using this method to optimize flight attitude and investigate the effect of wind (Jung et al., 2014; Jung et al., 2019). Considering that these studies have provided methods for acquiring or estimating the basic data (kinematics, GRF, and aerodynamics) required, a data-driven musculoskeletal simulation of in-field take-off seems feasible.

In this study, we aim to develop an effective approach for musculoskeletal simulation which considers both GRF and aerodynamic forces during the take-off phase of in-field ski jumping. Based on the musculoskeletal simulation, muscle activity and function were analyzed. Using this simulation model, it becomes feasible to study muscle activation and function during ski jumping, particularly in the situations where GRF and aerodynamic forces are challenging to measure.

## 2 Materials and methods

### 2.1 Trial protocol and video digitization

**FIGURE 1:**
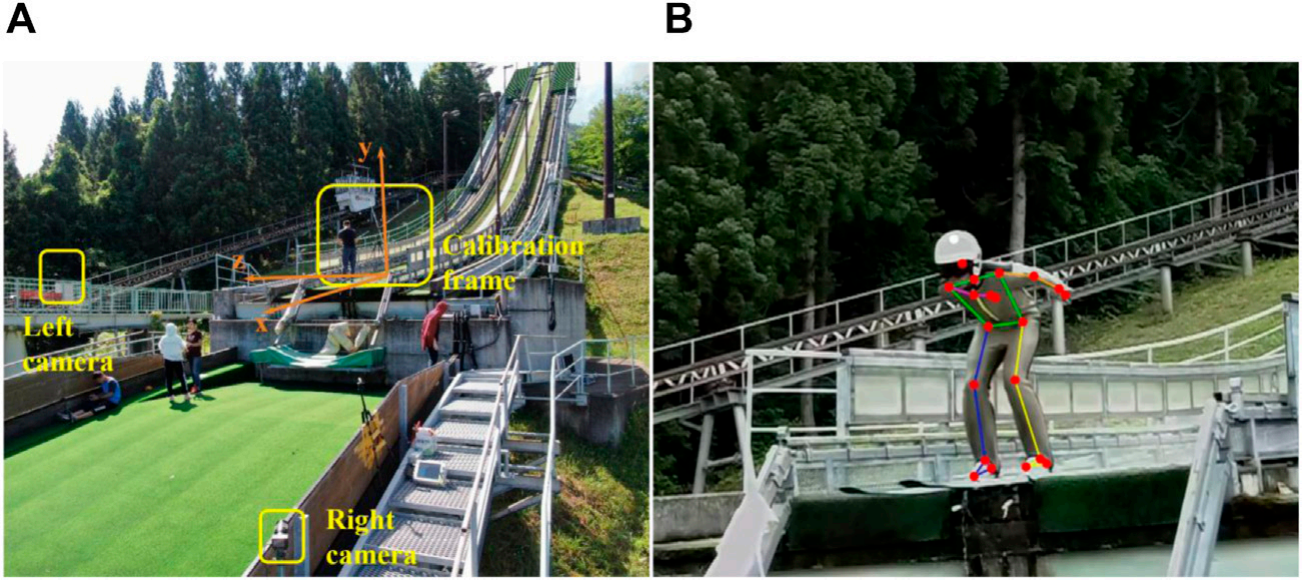
Acquisition of take-off kinematics. **(A)** Take-off trial equipment layout. **(B)** An example result of 2D automatic pose estimation aided with manual correction.

At the normal hill (hill size = 86 m) in Akita Japan, take-offs of eight male professional jumpers (23 ± 7.7 years old, 176 ± 4.3 cm, 62 ± 6.5 kg) from Chinese national ski jumping team were filmed after being informed. The trial protocol was reviewed and approved by the ethics committee of Capital University of Physical Education and Sports (approval number: 2022A41). Twenty-seven jumps (three or four jumps per participant) were recorded at 120 fps using two cameras positioning in front of the table edge. A 3D radial calibration frame (QFS-28 DLT Calibration Frame, HuiAnMing Sciences Co., Ltd., Beijing, China) was set up on the table and filmed for camera calibration. The equipment layout is shown in Figure 1A.

Videos from two cameras were synchronized based on the frame in which jumpers’ heels left the table edge. Video digitization was implemented through an automatic algorithm assisted with manual corrections. A 2D pose estimation algorithm (Motion-3D V1.2.5, Fastmove, Dalian, China) was used to identify 20 joint points of human body automatically (Figure 1B). Joint points with weak estimation accuracy were then corrected manually. Based on images of the calibration frame, 3D trajectories of 20 joint points were calculated by triangulation and will be used as the raw kinematics data for the following musculoskeletal simulation.

### 2.2 Model construction

It is critical for our simulation to use a suitable musculoskeletal model. The model developed by Lai et al. (Lai et al., 2017) was used as base model because of its’ refined lower extremity musculature. This model modified the range of knee flexion and properties of several muscles on the basis of the model published by Rajagopal et al., making it suitable for movements involving substantial knee and knee flexion, such as ski jumping take-off. In addition, since the external forces in this simulation would be calculated based on full-body kinematics, the model we used should also be capable of full-body movements tracking. Thus, the lumbar spine of the FBLS model (Raabe and Chaudhari, 2016), the head and neck of the MASI model (Cazzola et al., 2017), and the shoulder construction from the model developed by Blana et al. (Blana et al., 2008) were combined into the original Lai model to enable it to track full-body movements. While combining these models, inertial properties of each body were also adjusted to meet the mass distribution of a human body. Additionally, since the raw kinematic data was not enough to determine the movements of subtalar and metatarsal-phalangeal joints, these two joints were locked in the simulation.

The general model constructed above was then scaled based on athletes’ height and weight through scaling module of OpenSim (Scott L et al., 2007). Since these professional athletes have higher muscle strength than ordinary people, it is necessary to increase the muscle strength. The maximum isometric forces of all muscles were first doubled and then multiplied by a scaling factor based on the height and weight of each athlete (Handsfield et al., 2014).

### 2.3 GRF estimation

Due to equipment limitations, we were unable to measure the GRF directly during the take-off. However, we developed a general method based on inverse dynamics iteration to estimate the GRF from kinematic data. Given kinematics and kinetics, inverse dynamics solves the equations of motion and calculates generalized forces (Kuo, 1998), which include the compensating forces and torques directly acting on the pelvis to satisfy Newton’s second law, called residuals. These residuals represent dynamic inconsistencies between kinematics and kinetics, and consist of six components, namely, the components of force and torque along three orthogonal axes, denoted by $F_{x}^{res}$ , $F_{y}^{res}$ , $F_{z}^{res}$ , $M_{x}^{res}$ , $M_{y}^{res}$ , and $M_{z}^{res}$ . Assuming equal external loads on both legs, the GRF also consists of six components, denoted by $F_{x}^{l/r}$ , $F_{y}^{l/r}$ , $F_{z}^{l/r}$ , $M_{x}^{l/r}$ , $M_{y}^{l/r}$ , and $M_{z}^{l/r}$ respectively, where the superscripts l/r represent the left or right leg. By iteratively performing inverse dynamics and adding the residuals to the GRF, we were able to obtain a GRF that matches the kinematic data and minimizes residuals. Initially, all GRF components are set to 0 and applied at proximal second metatarsal, and inverse dynamic analysis is performed to calculate the residuals. Then, the following formula is performed to add the residual components to the GRF components of the left and right legs evenly and update the GRF:

$$\left\{\begin{array}{c} F_{x}^{l/r,s}=F_{x}^{l/r,s-1}+{\frac{1}{2}F}_{x}^{res,s}\\ F_{y}^{l/r,s}=F_{y}^{l/r,s-1}+{\frac{1}{2}F}_{y}^{res,s}\\ F_{z}^{l/r,s}=F_{z}^{l/r,s-1}+{\frac{1}{2}F}_{z}^{res,s}\\ M_{x}^{l/r,s}=M_{x}^{l/r,s-1}+{\frac{1}{2}M}_{x}^{res,s}\\ M_{y}^{l/r,s}=M_{y}^{l/r,s-1}+{\frac{1}{2}M}_{y}^{res,s}\\ M_{z}^{l/r,s}={M_{z}^{l/r,s-1}+\frac{1}{2}M}_{z}^{res,s} \end{array}\right.$$ (1)

where superscript s is the index of iterative steps. In each iteration, the mean root mean square (RMS) of residuals through the time period of simulation is calculated. Finally, when the mean RMS is less than the threshold 0.001 (N for forces and Nm for torques), the residual is considered close enough to 0 and the GRF is output as result.

In order to verify this method under standard in-lab motion capture trial procedure, an imitation jump trial was implemented. After being informed the trial procedure, three healthy adult men (29 ± 2.2 years old, 172 ± 0.04 cm, 83 ± 2.83 kg) from local college agreed to participate. The trial protocol was reviewed and approved by the ethics committee of Capital University of Physical Education and Sports (approval number: 2022A41). Participants were asked to perform 3 jumps each. Thirty-nine reflective markers were attached to multiple anatomical landmarks all over the body. Raw marker kinematics data were collected by a 12-camera motion capture system (Optitrack Prime 22, NaturalPoint, Corvallis, United States) with 200 fps, while the GRF were collected synchronously with 1,000 Hz by two force plates (Multicomponent Force Plate 9260AA, Kistler, Winterthur, Switzerland). GRF estimated by the above method was compared with the result of force plates.

### 2.4 Aerodynamic force calculation

The high-speed nature of ski jumping makes the aerodynamic effects significant enough to be considered in the simulation. In our case, aerodynamic forces during take-off were estimated based on body kinematics and CFD simulations.

**FIGURE 2:**
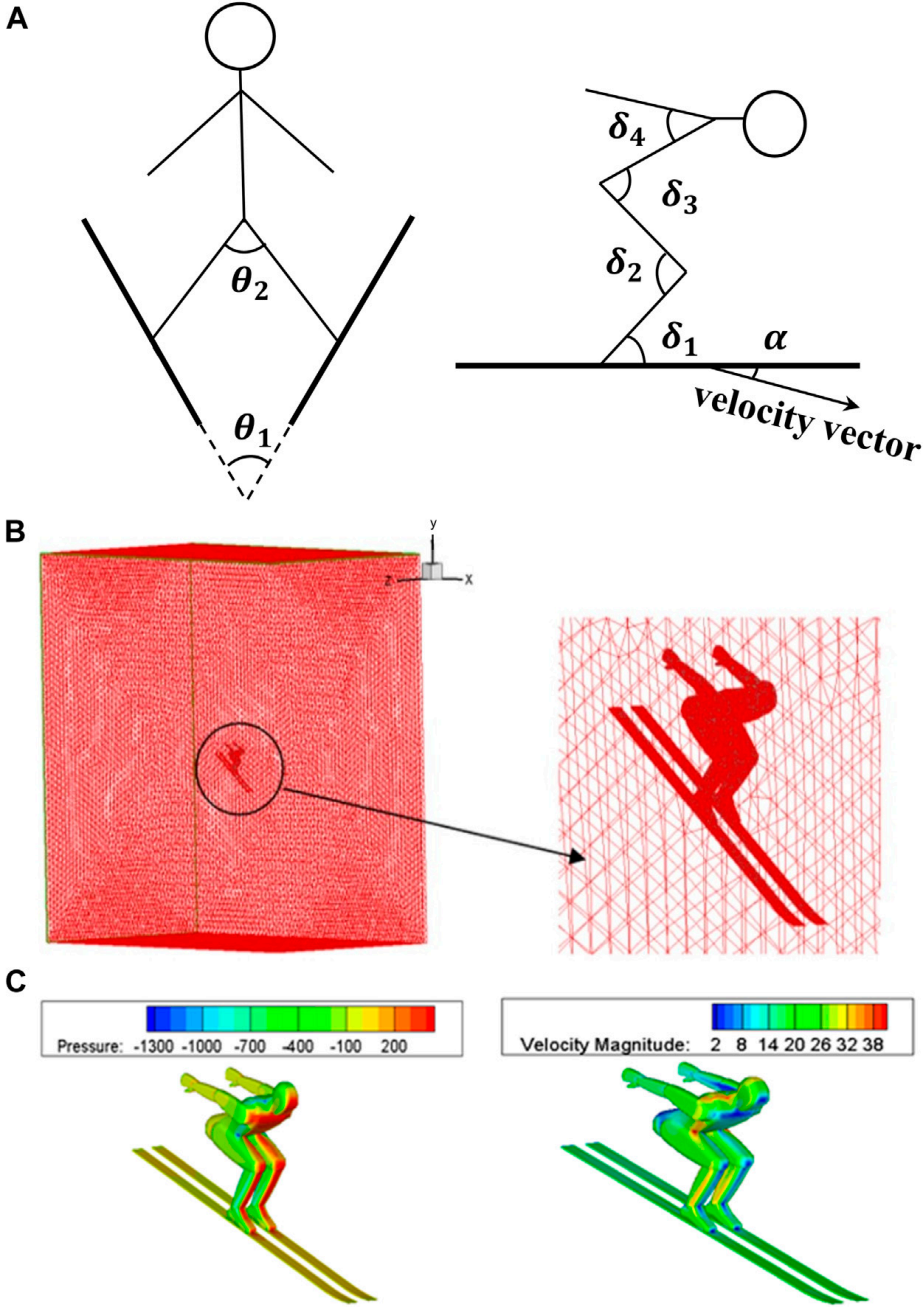
Schematic diagram of CFD simulation setup. **(A)** Posture angles definition. **(B)** Flow field meshing. **(C)** Boundary condition settings.

CFD simulations were carried out under various possible ski jumping postures. Seven representative posture angles with a significant impact on jumper aerodynamic properties were selected (Figure 2A, including the angle between legs $\theta_{2}$ , the angle between skis $\theta_{1}$ , the angle between arms and trunk $\delta_{4}$ , the angle between trunk and thighs $\delta_{3}$ , knee angle $\delta_{2}$ , ankle angle $\delta_{1}$ , and the angle of attack α ). Based on jumpers’ possible postures during the take-off and early flight phases, several values were selected for each angle to form a total of 278 postures (Table 1). Under each posture, geometric models of the jumper and skis were constructed using Gambit (V 2.4.6). The jumper model was 1.70 m high. The size of the skis was 2.57 × 0.22 m. Triangular unstructured surface grid was generated over the model surface. A tetrahedral unstructured grid was then generated in the computational domain with a size of 15 m (x) × 15 m (y) × 8 m (z) (Figure 2B). There were about 1.5 million grids in total. No-slip wall, velocity inlet, and pressure outlet boundary conditions (Figure 2C) were set up in Fluent (V 16.1). The inlet flow velocity was set to be 23 m/s (according to FIS Certificates of the jumping hill). The incompressible flow model was adopted. The mathematical model adopts the Reynolds-averaged N-S equation and the k-ε turbulence model. Using the first-order upwind style for spatial discretization, the SIMPLE algorithm was used to iteratively compute until convergence. Aerodynamic forces and moments were calculated by integrating the pressure field over the model surface. The model was also divided into 10 parts: left and right calves, thighs, forearms, and upper arms as well as trunk and head, to integrate separately. All aerodynamic forces and moments were imported into a ski jumping aerodynamic database.

**TABLE 1:** The values of posture angles.

Posture angles	Value (°)				
	Take-off			Early flight	
$\delta_{2}$	90	120	150	150	180
$\delta_{3}$	20, 50, 80, 110	20, 50, 80, 110, 140	20, 50	80, 110, 140, 180	80, 110, 140, 180
$\theta_{2}$	0	0	0	0, 30	0, 30
$\theta_{1}$	0	0	0	0, 45	0, 45
$\delta_{4}$	0	0	0	0, 30	0, 30
$\delta_{1}$	30, 90	30, 90	30, 90	30, 90	30, 90
α	0	0	0	0, 30	0, 30

**FIGURE 3:**
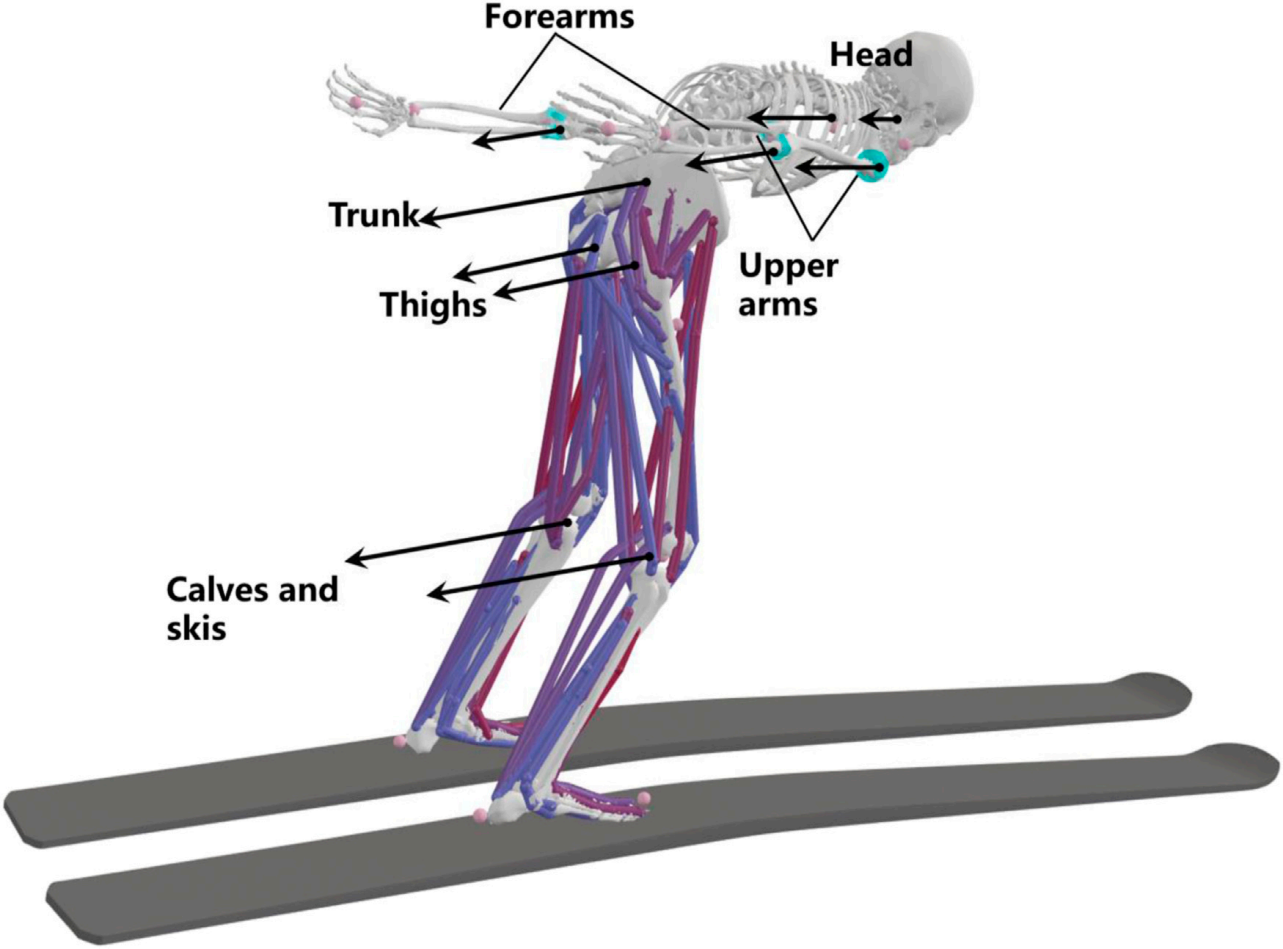
Schematic of the aerodynamic forces being applied to the model.

Aerodynamic forces were estimated by matching the measured body motion sequences with the postures in the database. The above posture angles during the take-off were calculated based on model and marker set kinematics. Wherein, ski postures were calculated by markers on heels and toes. The angles in each frame were linearly interpolated in the multidimensional posture space to calculate the corresponding aerodynamic force. Flow velocity and body size were set as constant values in the above CFD simulations. To reflect these effects, the following empirical formula was used:

$$F_{AD}=\frac{\rho }{2}cAw^{2}$$ (2)

where $F_{AD}$ is the aerodynamic force, ρ the air density, c the aerodynamic coefficient which is related to the body postures and clothing, A the cross-sectional area, and w the speed of the body. A is assumed to be linearly related to the height of the athlete. Therefore, the aerodynamic forces calculated above were scaled linearly by height and quadratically by velocity. The resulting aerodynamics were used as external loads in musculoskeletal simulations as shown in Figure 3.

### 2.5 Musculoskeletal simulation

**FIGURE 4:**
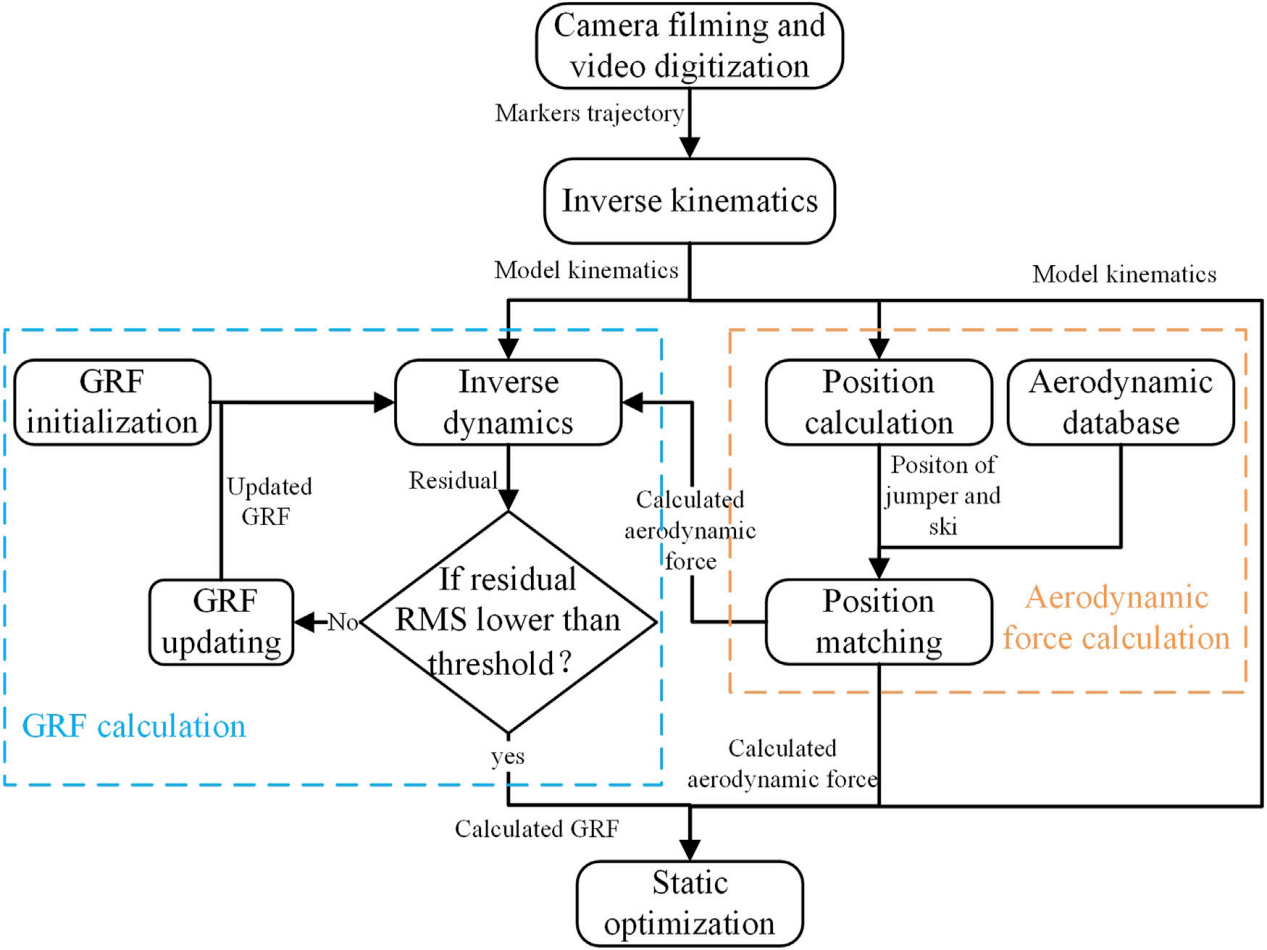
Schematic diagram of data processing workflow.

**FIGURE 5:**
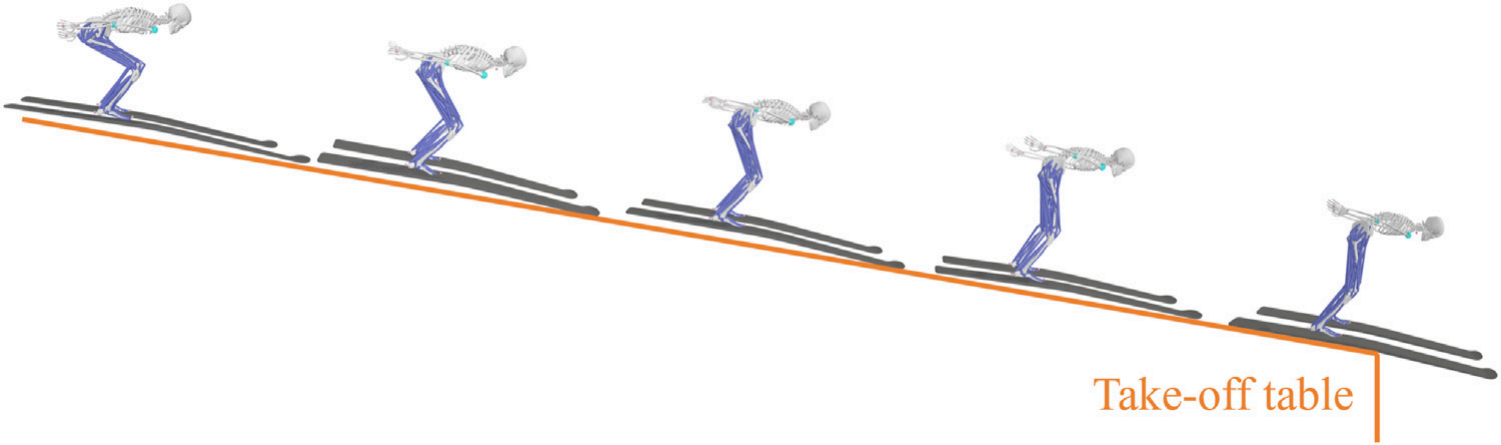
An example of musculoskeletal model motion sequence.

All data processing procedures were implemented through OpenSim 4.1 (Stanford University, Stanford, United States) shown in Figure 4. Movements within 0.1s before release were simulated. Firstly, full-body kinematics were calculated from joint points trajectories through inverse kinematics (Lu and O'Connor, 1999). An example of model motion sequence from inverse kinematics is shown in Figure 5. Then, GRFs and aerodynamic forces were calculated through the method established above. The GRF components in the medial-lateral direction and all moment components were ignored, which allows $F_{z}^{l/r}$ , $M_{x}^{l/r}$ , $M_{y}^{l/r}$ , and $M_{z}^{l/r}$ being considered equal to zero. $F_{y}^{l/r}$ was calculated iteratively by inverse dynamics. $F_{x}^{l/r}$ was the friction between the skis and table with a friction coefficient of 0.018 (calculated by a friction angle of 1° according to the regulations of the International Ski Federation) (Gasser, 2018). Finally, muscle activation and force were calculated through static optimization using a criterion of minimizing the sum of squared muscle activation (Thelen and Anderson, 2006). The torque contributions of these muscles to the lower extremity joints were further calculated by multiplying muscle force by moment arm.

## 3 Results

### 3.1 GRF estimation verification

**FIGURE 6:**
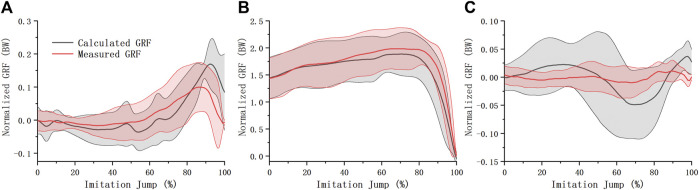
GRF of imitation jump. Estimated imitation jump GRF (mean ± S.D.) were compared with measured one in the anterior-posterior **(A)**, vertical **(B)**, and medial-lateral **(C)** direction.

Measured and estimated GRFs of simulated jumps were normalized to body weight (Figure 6). They were similar in the anterior-posterior and vertical directions, while the differences were larger in the medial-lateral direction. The mean root mean square error (RMSE) between the measured and estimated GRFs was 39.92 N, 106.41 N, and 19.44 N in the anterior-posterior, vertical, and medial-lateral directions, respectively.

### 3.2 Take-off kinematics and kinetics

The mean RMSE between the coordinates of the simulated markers derived from inverse kinematics and the measured one was 3.13 cm, which was slightly larger than the reported result using a marker-based motion capture system (1.38–2.03 cm) (Glover et al., 2021).

**FIGURE 7:**
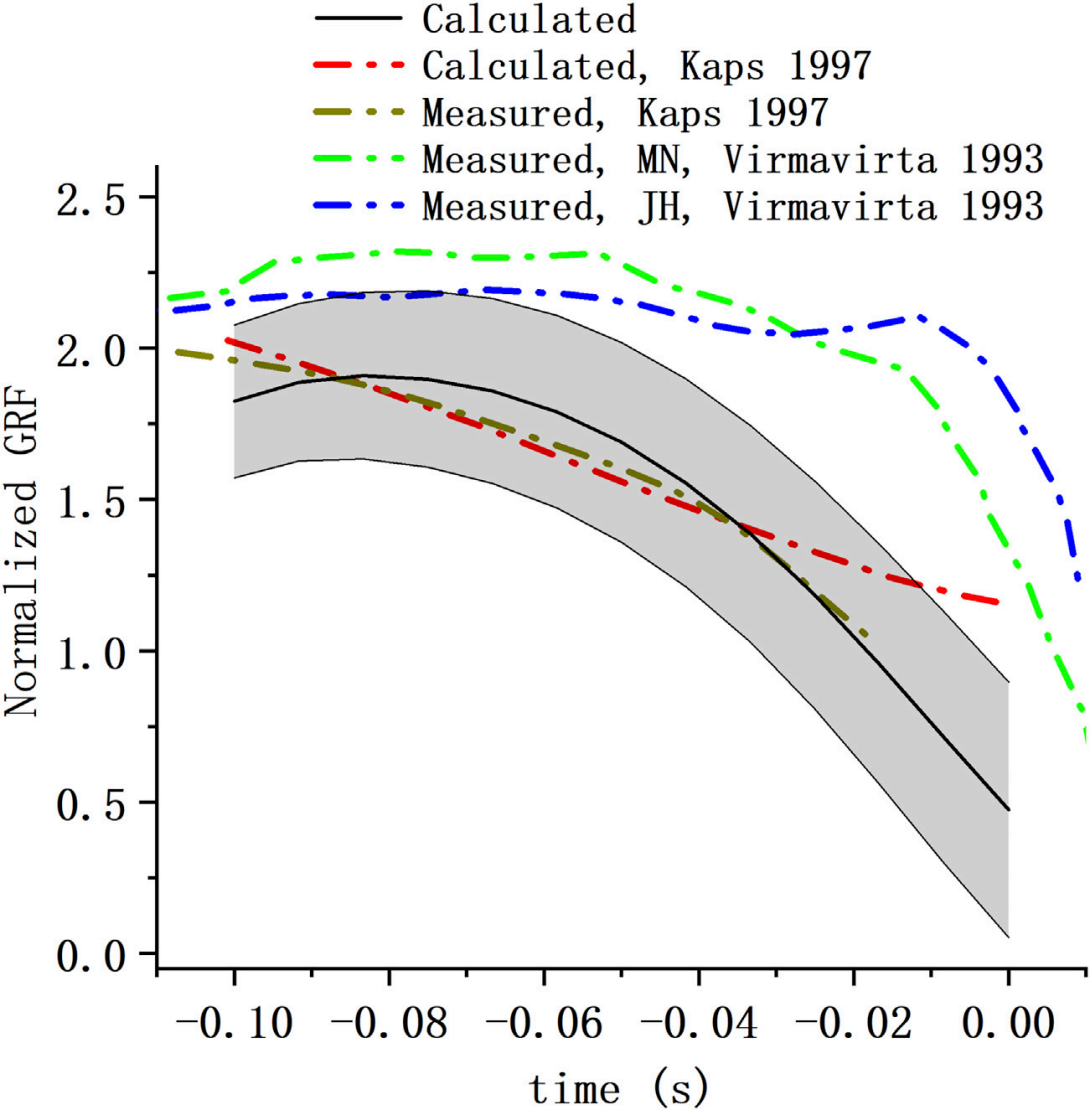
Take-off GRF of hill jump. Take-off GRF perpendicular to the table from our calculation (mean ± S.D.) were compared with GRF from the study of Kaps et al. (Kaps et al., 1997) and that of Virmavirta and Komi (Virmavirta and Koml, 1993b) (MN and JH represent the two athletes tested in the study).

Take-off GRF (normalized to body weight) component perpendicular to the table from our calculation was reported and compared with the GRF from 2 previous studies (Virmavirta and Koml, 1993b; Kaps et al., 1997) (Figure 7). The take-off GRFs all share a similar trend, that is, decrease as the take-off process. The GRF from our estimation reduced more rapidly than that of Virmavirta and Komi.

**FIGURE 8:**
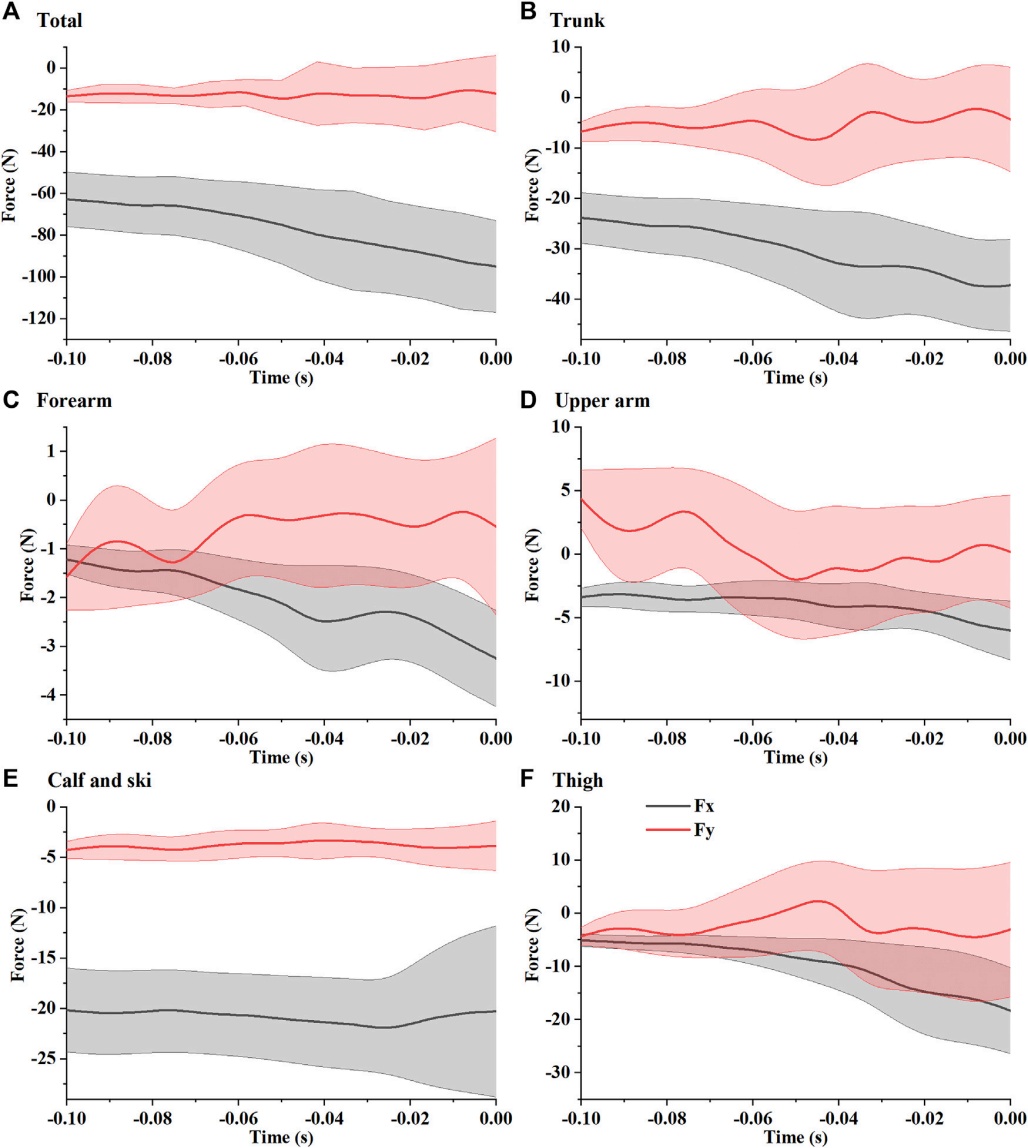
Calculated aerodynamic force (mean ± S.D.) for the whole body **(A)**, trunk **(B)**, forearm **(C)**, upper arm **(D)**, calf and ski **(E)**, and thigh **(F)**.

The calculated take-off aerodynamic resultant force (mean ± S.D.) was shown in Figure 8A, where $F_{x}$ and $F_{y}$ represent the components of the force in the *x* and *y* directions (the coordinate system is defined in Figure 1A), respectively. The drag force along the direction of the platform gradually increased from 60 N to about 90 N, while the drag force perpendicular to the table was about 10 N–20 N. The aerodynamic forces (mean ± S.D.) acting on each body part are shown in Figures 8B–F. Aerodynamic force acts on the trunk and on calves and skis primarily.

### 3.3 Muscle activity

**FIGURE 9:**
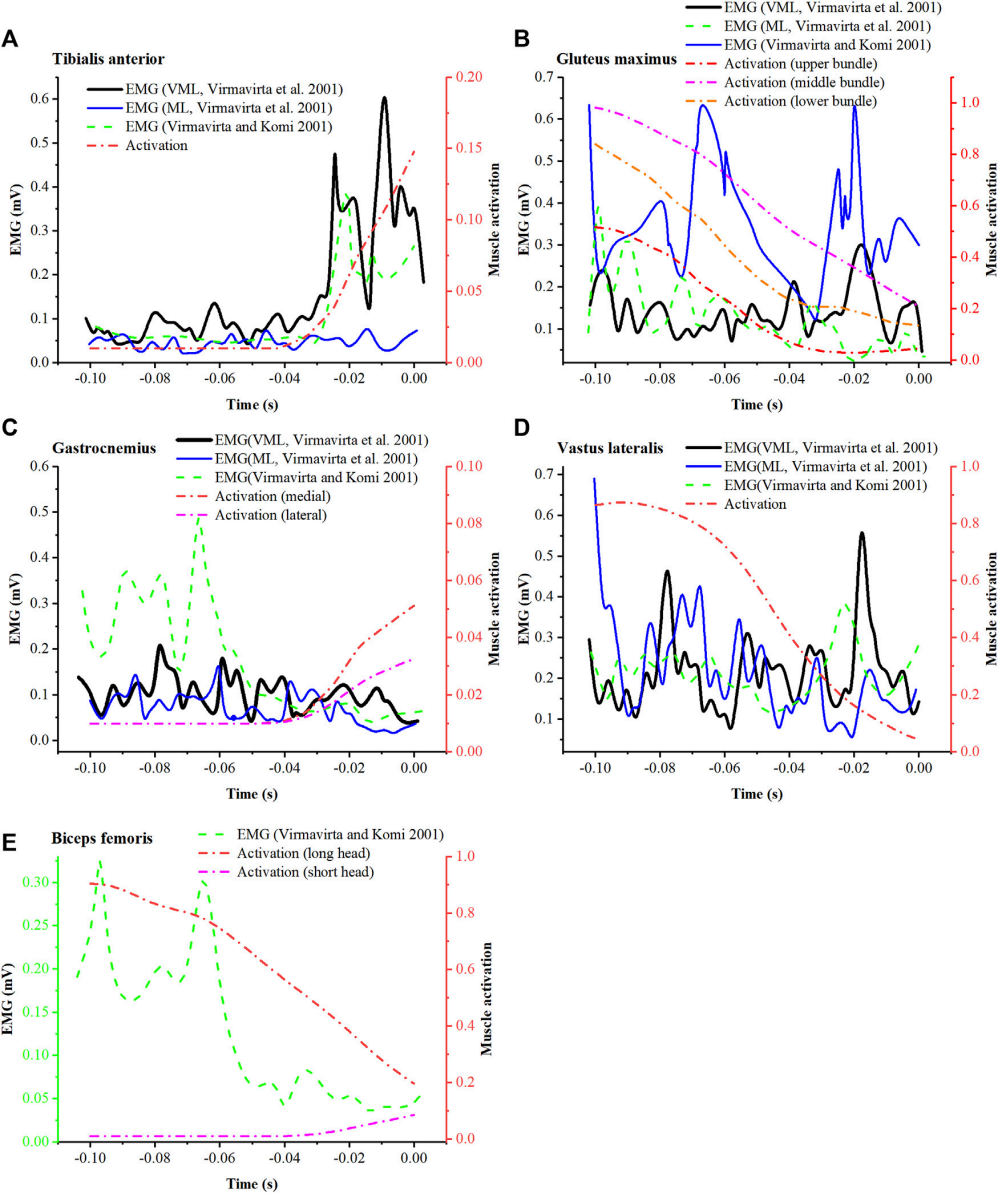
Comparison between simulated muscle activation (mean) and EMG from the research of Virmavirta et al. (2001) (VML and ML represent 2 jumpers in the study) and the research of Virmavirta and Komi (2001) for tibialis anterior **(A)**, gluteus maximus **(B)**, gastrocnemius **(C)**, vastus lateralis **(D)**, and biceps femoris **(E)**.

Simulated mean muscle activation was reported and compared with EMG (Figure 9) from study of Virmavirta et al. (Virmavirta et al., 2001) (EMG of jumper VML and ML on hill K-90) and that of Virmavirta and Komi (Virmavirta and Komi, 2001) (mean EMG of ten jumpers on hill K-100). The simulated activation of the tibialis anterior showed a similar trend to both the EMG data of the jumper VML and the mean EMG data of ten athletes (Figure 9A). Similarly, the simulated activation of the gluteus maximus was similar to the EMG data of jumper ML (Figure 9B), and the activation of the biceps femoris long head was similar to the mean EMG of ten athletes (Figure 9E). As for other muscles, the simulated activation was less consistent with EMG.

**FIGURE 10:**
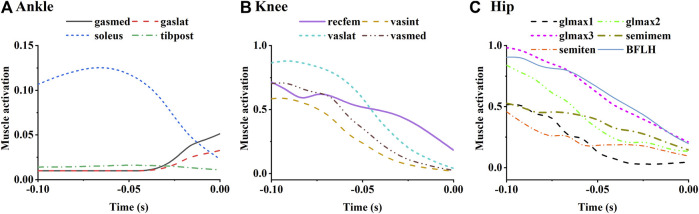
Simulated mean muscle activation of the ankle **(A)**, knee **(B)** and hip **(C)** extensors. Ankle plantar flexors include medial gastrocnemius (gasmed), lateral gastrocnemius (gaslat), soleus, and tibialis posterior (tibpost). Knee extensor muscles include rectus femoris (recfem), vastus lateralis (vaslat), vastus medialis (vasmed), and vastus intermedius (vasint). Hip extensors include gluteus maximus, semimemem (semiten), and biceps femoris long head (BFLH). Gluteus maximus is constructed in the musculoskeletal model as upper (glmax1), middle (glmax2), and lower (glmax3) bundles.

Simulated activation of selected lower limb muscles was shown in Figure 10. Muscles with the function of lower limb extension were selected and grouped as ankle plantar flexors, knee extensors, and hip extensors to plot. Activation level of the ankle plantar flexors was relatively low (Figure 10A). The soleus was activated until the end of the take-off, where the gastrocnemius began to activate slightly. The activation level of quadriceps decreased with take-off progress (Figure 10B). Similarly, the activation level of the hip extensors also decreased with take-off progress, among which lower bundles of gluteus maximus and biceps femoris long head showed higher activation level than the others (Figure 10C).

**FIGURE 11:**
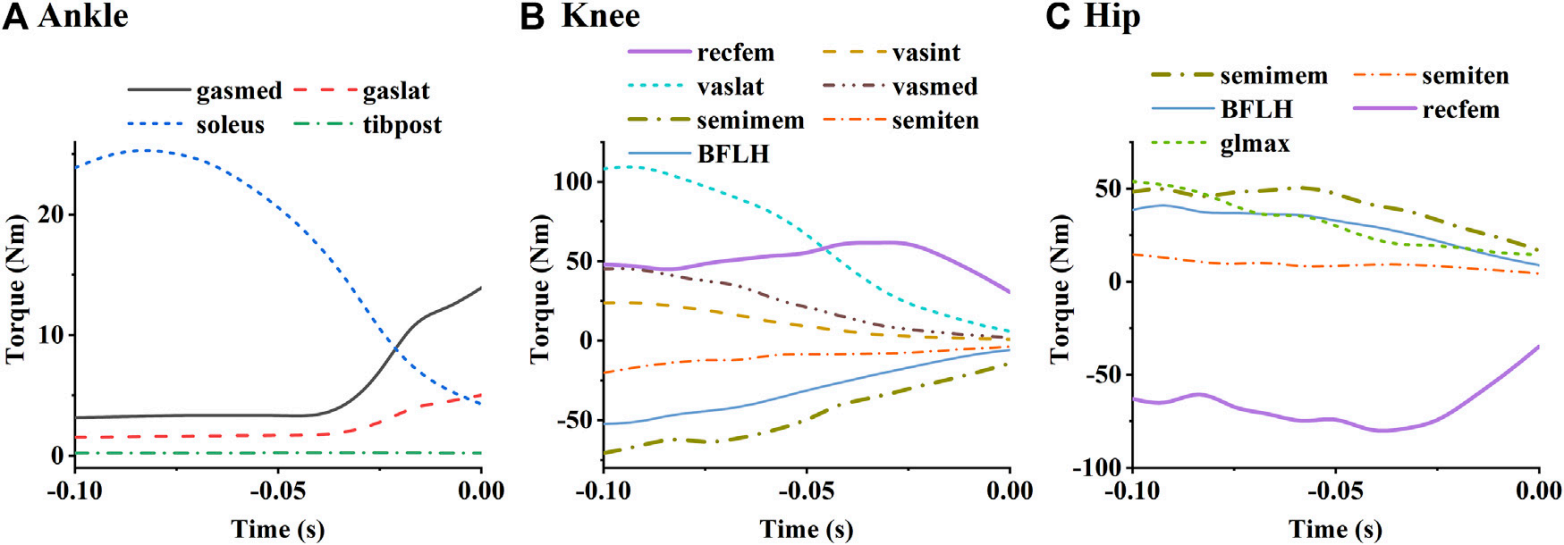
Simulated mean torques of each muscle to the ankle **(A)**, knee **(B)**, and hip **(C)**. The labels in the figure are the same as those in Figure 8.

The torque contributions of the lower extremity muscles to the ankle, knee, and hip joints are shown in Figure 11. The relative magnitudes of the extension torques produced by the ankle plantar flexors and knee extensors on their corresponding joints (Figures 11A,B) were approximately consistent with the relative magnitudes of their activation levels. As for the hip joint (Figure 11C), the extension torque produced by the long head of the biceps femoris, gluteus maximus, and semimembranosus were similar, although the activation level of the biceps femoris was higher than the others. In addition, the biceps femoris long head and semimembranosus were found to generate large flexion torques on the knee, while the rectus femoris produces large flexion torque on the hip.

## 4 Discussion

In this study, we applied musculoskeletal simulation to ski jumping take-off. With newly developed methods, GRFs and aerodynamic forces were calculated and used in the simulation. The GRF estimation method was verified through in-lab trial and showed results close to the measurements. Calculated aerodynamic forces were also reported. Simulated GRFs and muscle activation were compared with the results from previous studies to demonstrate the validity of this simulation. The simulation results showed high activation levels and large extension torque contributions for the soleus, vastus lateralis, gluteus maximus, semimembranosus, and biceps femoris long head.

The deviation between the estimated and measured GRFs in the medial-lateral direction during the simulated jump was large (Figure 6C), probably because the GRFs in this direction were relatively small and were covered by errors. Previous studies of Data-driven GRF estimation to the take-off (Kaps et al., 1997; Logar and Munih, 2015; Fritz et al., 2019; Nam et al., 2023) share the same theoretical basis as ours, that is, the inverse dynamics analysis for multibody models. The computational accuracy of this type of method depends on the quality of the raw kinematics data and how well the model is able to track the kinematics. Compared with two-dimensional models in previous studies, the three-dimensional model we used is able to capture the potential asymmetry of body movements. By taking coupled joint motion of shoulders, neck and spine into account, the model we used has a better ability to track the upper body motion than simplified head-torso models previously reported. Additionally, the GRF estimation pipeline was merged with inverse dynamics analysis of Opensim, making the estimated GRF can be conveniently used in subsequent musculoskeletal simulations.

Rigorous musculoskeletal simulation validation requires agreement between simulated muscle activation and measured EMG of the same movements. However, due to the limitations of the ski jump field environment, it was challenging to set up laboratory-grade equipment on-site. Additionally, considering the safety concerns in ski jumping, athletes were not permitted to wear any measurement devices. We were unable to perform synchronous motion capture and EMG testing. Therefore, simulated muscle activation can only be assessed by comparison with measured EMG from previous studies. Overall low agreement between simulated muscle activation and measured EMG was observed (Figure 9). These differences could be caused by trial condition differences, individual motion differences, and simulation errors. Differences in hill size may not affect the comparison significantly, as the data being compared are close in hill size (K-86, K-90, and K-100). The inconsistency between activation and EMG could be primarily caused by individual differences, since Virmavirta et al. (Virmavirta et al., 2001) reported significant inter-individual differences in muscle activation patterns under the same test conditions. In addition, the errors of kinetic and kinematic data may also lead to unrealistic muscle activity. Nonetheless, similarities between activation and EMG in the tibialis anterior, gluteus maximus, and biceps femoris long head (Figures 9A,B,E) demonstrate the ability of musculoskeletal simulations to characterize the activity of some muscles.

Muscle activation levels are influenced by muscle function, muscle strength, as well as by force-velocity and force-length effects determined from body kinematics. As the muscles with high strength are more likely to be recruited, soleus among ankle plantar flexors, vastus lateralis among knee extensors, as well as biceps femoris long head, gluteus maximus, and semimembranosus among hip extensors showed high levels of activation (Figure 10). Increasing the strength of these muscles may contribute to greater joint extension torque and higher take-off speed. Biarticular muscles (acting as extensors in one joint and flexors in the other) tend to have relatively low activation levels, such as the gastrocnemius and semitendinosus. However, the rectus femoris, the long head of the biceps femoris, and the semimembranosus, which also function in 2 joints, showed high activation levels for their torque contributions are necessary for motion execution (Figures 11B,C). Activity of these muscles can potentially limit athletes’ take-off speed.

There are some limitations in this study. Firstly, the mean RMSE between the measured marker trajectories and the simulated ones indicates that the overall kinematic error of the marker-less motion capture system is slightly larger than that of marker-based motion capture systems such as Vicon or Qualisys. It is possible to further improve kinematic accuracy through personalized anthropometric measurements, better performing joint point estimation systems, or higher resolution cameras. The effect of less precise raw kinematics on subsequent dynamics and muscle state simulations remains to be further investigated. Secondly, the musculoskeletal simulations were not rigorously validated in this study, which limited the explanatory power of our results for take-off muscle action. Simulated muscle activation pattern needs to be further verified by comparison with the synchronously measured EMG. Finally, this is a group study of multiple jumpers. It can only reflect the common characteristics of take-off. Future research may focus on individual take-off dynamics, muscle activity and their impact on ski jumping performance.

## 5 Conclusion

This is a preliminary study that implemented musculoskeletal simulation of professional jumpers during take-off considering aerodynamic forces for the purpose of approach validation and muscle action analysis. The simulated GRFs were similar to both in-lab GRFs from force plates and in-field GRFs from previous studies. Although there were inconsistencies between the results of some muscle activation in the simulation and EMG from previous studies in general, it is worth noting that the activation of the tibialis anterior, gluteus maximus, and long head of the biceps femoris was similar to specific EMG results. This approach may be further used to explore the musculoskeletal dynamics of other ski jumping movements.

## Data Availability

The original contributions presented in the study are included in the article/Supplementary Material, further inquiries can be directed to the corresponding authors.
